# Clinical Application of Surface Plasmon Resonance-Based Biosensors for Fetal Fibronectin Detection

**DOI:** 10.3390/s120403879

**Published:** 2012-03-26

**Authors:** Chen-Yu Chen, Chia-Chen Chang, Chun Yu, Chii-Wann Lin

**Affiliations:** 1 Institute of Biomedical Engineering and College of Medicine, National Taiwan University, No. 1, Roosevelt Road, Taipei 10617, Taiwan; E-Mails: d96548019@ntu.edu.tw (C.-Y.C.); ccchang.ibme@gmail.com (C.-C.C.); f93548059@ntu.edu.tw (C.Y.); 2 Department of Obstetrics and Gynecology, Mackay Memorial Hospital, No. 92, Section 2, Zhongshan N. Road, Taipei 10449, Taiwan; 3 Mackay Medicine, Nursing and Management College, No. 92, Shengjing Road, Taipei 112, Taiwan

**Keywords:** fibronectin (fFN), surface plasmon resonance (SPR), preterm birth, biosensor

## Abstract

Preterm birth is the leading cause of perinatal morbidity and mortality. Fetal fibronectin (fFN), a glycoprotein in the extracellular matrix of the amniotic membranes, is the most powerful biomarker for predicting the risk of preterm birth. Biosensors using the surface plasmon resonance (SPR) response are potentially useful in quantitatively measuring molecules. We established a standard calibration curve of SPR intensity against fFN concentration and used the SPR-based biosensor to detect fFN concentrations in the cervicovaginal secretions of pregnant women between 22 and 34 weeks of gestation. The calibration curve extends from 0.5 ng/mL to 100 ng/mL with an excellent correlation (R^2^ = 0.985) based on standard fFN samples. A cutoff value of 50 ng/mL fFN concentration in commercial ELISA kits corresponds to a relative intensity of 17 arbitrary units (a.u.) in SPR. Thirty-two pregnant women were analyzed in our study. In 11 women, the SPR relative intensity was greater than or equal to 17 a.u., and in 21 women, the SPR relative intensity was less than 17 a.u. There were significant differences between the two groups in regular uterine contractions (*p* = 0.040), hospitalization for tocolysis (*p* = 0.049), and delivery weeks (*p* = 0.043). Our prospective study concluded that SPR-based biosensors can quantitatively measure fFN concentrations. These results reveal the potential utility of SPR-based biosensors in predicting the risk of preterm birth.

## 1. Introduction

Preterm birth is defined by the World Health Organization as delivery at less than 37 gestational weeks [1]. It is the main cause of perinatal morbidity and mortality, with an incidence of 5 to 13% in developed countries, and it accounts for nearly 70% of neonatal deaths and 50% of neurological disabilities [2–5]. Early detection of preterm birth is difficult because the initial signs and symptoms are often obscure and may be mimicked by normal pregnancies. Therefore, it is essential to find biomarkers to predict the risk of preterm birth. Previous studies have revealed that various biomarkers in cervicovaginal secretions are related to preterm birth, such as fetal fibronectin (fFN), interleukin-6 (IL-6), monocyte chemotactic protein-1 (MCP-1), phosphorylated insulin-like growth factor binding protein-1 (phIGFBP-1), and matrix metalloproteinase-8 (MMP-8) [6–10]. Of these biomarkers, the most powerful is fFN because of its high negative predictive value: only 1% of pregnant women with a negative fFN result deliver within the next week [2,11–13].

fFN is a glycoprotein found in the extracellular matrix of the amniotic membranes [14]. It can be distinguished from other human fibronectin proteins because of the specific III-CS (connecting segment) epitope, which is recognized by the FDC-6 monoclonal antibody in immunohistochemical staining [15,16]. fFN is generally found in cervicovaginal secretions until 22 gestational weeks; it then diminishes between 22 and 34 gestational weeks and increases again near term in normal pregnancies. However, preterm labor induces the release of fFN into the ectocervix or posterior vaginal fornix. The putative mechanisms of fFN release are either the mechanical force caused by uterine contractions leading to choriodecidual separation or inflammation resulting from subclinical bacterial infection that degrades the choriodecidual interface [17,18]. Numerous studies have demonstrated the clinical usefulness of fFN in predicting the risk of preterm labor [12,13,19–21]. The FDA has approved an fFN enzyme-linked immunosorbent assay (ELISA) and a lateral flow, solid-phase immunochromatographic assay (Rapid fFN Cassette) (TLi system, Adeza Biomedical Corporation, Sunnyvale, CA, USA) to predict the risk of preterm birth. In these assays, an fFN concentration greater than or equal to 50 ng/mL is defined as a positive result and shows a higher risk of preterm birth. However, these two conventional methods have some drawbacks. The ELISA method is time consuming, and its results are influenced by the color signal intensity, whereas the Rapid fFN Cassette is not a quantitative method. Therefore, a quantitative, label-free, and easy-to-perform assay is required.

In recent years, a biosensor using the response of surface plasmon resonance (SPR) was introduced [22–24]. SPR is an electromagnetic reaction of surface plasmons at the metal–dielectric interface of biosensors. In theory, when the analyte binds the ligand on the metal film, the interfacial architecture changes, and surface plasmons are excited by the light beam. We can measure the change of the resonant angle and determine the concentration of biomolecules of interest. SPR intensity measurement effectively enhances the accuracy of many spectroscopic measurements because it not only has a highly sensitive response in biomolecular interactions, but it also allows real-time monitoring in label-free environments. We performed a prospective study to detect fFN concentrations in the cervicovaginal secretions of pregnant women between 22 and 34 gestational weeks using SPR-based biosensors and attempted to predict the risk of preterm birth using these fFN measurements.

## 2. Materials and Methods

### 2.1. Sample Collection

We performed the study in the delivery room of the Mackay Memorial Hospital from October 2009 to May 2010. The cervicovaginal secretions of pregnant women who complained of low abdominal pain or uterine contractions between 22 and 34 gestational weeks were collected by speculum examination before performing any other transvaginal procedures such as vaginal ultrasound and endocervical culture, which may rub the cervical mucosa and thus interfere with sample collection. We slightly inserted the tip of a sterile cotton swab to the posterior fornix of the vagina and rotated the swab for 10 s to absorb secretions. After removing the cotton swab, we inserted the tip of the cotton swab into a tube containing 3 mL of phosphate buffered saline (PBS) and mixed vigorously for 10 s [25]. We then sent the fFN samples to the laboratory for SPR detection. The study was approved by the Mackay Memorial Hospital Institutional Review Board (IRB #09MMHIS056).

### 2.2. Fabrication of SPR Chip Substrates

Two different sensing substrates, Au (50 nm) and Cr (2 nm), were used as the electroplating materials. Standard glass microscope slides (SF-10, Schott Glass) were used as base substrates. The slides were first cleaned with piranha solution (H_2_SO_4_:H_2_O_2_ = 3:1) for 10 min and then rinsed three times with deionized water. The slides were then dried with nitrogen gas. Au and Cr films were deposited on the slides by an electron beam evaporator at a vacuum level of about 3 × 10^−6^ Torr.

### 2.3. Preparation of the Biosensor Surface

First, the gold-coated slide was immersed into an 8-mercaptooctanoic acid (8-MOA) solution with a concentration of 10 mM at room temperature (approximately 25 °C) for 30 min [22]. Then, the chemically immobilized surface was activated using 400 mM 1-ethyl-3-(3-dimethylaminopropyl)- carbodiimide (EDC)/100 mM N-hydroxysuccinimide (NHS) for covalent bond formation in 15 min at room temperature. Next, the gold film chip was soaked in 50 μg/mL protein G solution, which was fixed on the gold surface for 15 min. Then, 0.5 μg/mL rabbit monoclonal fFN antibody (Abcam, ab32419), reacting with a recombinant full length protein, was fixed onto the surface for 30 min. Finally, the surface of the chip was blocked with 1% bovine serum albumin (BSA) for 10 min. All steps were performed at room temperature.

### 2.4. SPR Measurement

SPR signal detection was accomplished using a GWC SPR Imager (GWC Technologies Inc. Madison, WI, USA). All measurements were performed at a fixed incident angle close to the SPR angle. First, PBS was allowed to flow over the sensing surface until a stable baseline was obtained. This step was followed by the introduction of the fFN solution onto the antibody-immobilized surface at a rate of 40 μL/min for 10 min. Next, unbound fFN antigens were washed out with PBS for another 10 min to achieve equilibrium. The measured value of the SPR reflectivity is expressed in arbitrary units (a.u.).

### 2.5. Data Analysis

Statistical analysis was performed with Student's *t* test for continuous variables and the *X*^2^ test for categorical data. For continuous variables, the results are presented as the mean ± standard deviation. *p* < 0.05 was considered statistically significant. A calibration curve was established using Sigma Plot software version 10.0 and fitted with the four-parameter logistic equation, which can be expressed as [26]:

$$\text{Y}={\text{I}}_{\text{L}}+({\text{I}}_{\text{H}}-{\text{I}}_{\text{L}})/[1+{\left(\text{C}/{\text{C}}_{1/2}\right)}^{\text{s}}]$$

in which Y represents the SPR intensity signal, I_L_ is the minimal SPR relative intensity, I_H_ is the maximal SPR relative intensity, C is the analyte concentration, C_1/2_ is the inflexion point concentration corresponding to the half-maximal SPR relative intensity, and S is the slope at the inflection point of the calibration curve.

A receiver-operating characteristic (ROC) curve was constructed to seek the optimum cut-off point of delivery week prediction for the group with higher SPR relative intensity. The optimum cut-off point was defined as the closest point on the ROC curve to the point (0, 1), that is, a false positive rate of zero and a sensitivity of 100%. The area under the curve (AUC) and 95% confidence interval (CI) were calculated. Statistical analysis was performed using the Statistical Package for the Social Sciences (SPSS), version 18.0.

## 3. Results and Discussion

### 3.1. Calibration Curve Establishment

**Figure 1.**
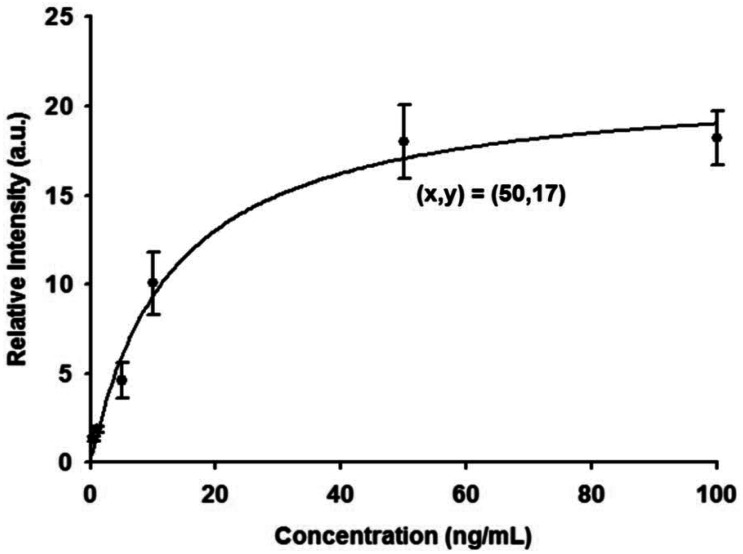
Calibration curve of SPR intensity against the concentrations of fetal fibronectin (fFN) (0.5, 1, 5, 10, 50, and 100 ng/mL), with a correlation coefficient (R^2^) of 0.985. The SPR intensity corresponding to the fFN ELISA critical concentration of 50 ng/mL was 17 a.u.

A calibration curve of SPR responses was first established from triplicate measurement of fFN antigen concentrations ranging between 0.5 ng/mL and 100 ng/mL, as shown in Figure 1. SPR relative intensities were measured with concentrations of 0.5, 1, 5, 10, 50, and 100 ng/mL of fFN antigen over a chip fixed with 0.5 μg/mL fFN antibody. The investigational data revealed excellent agreement when fitted to the calibration curve, with a correlation coefficient (R^2^) of 0.985. The SPR intensity corresponding to the critical concentration of 50 ng/mL in the fFN ELISA assay for the risk evaluation of preterm birth was 17 a.u,. The SPR intensity response increased with increasing fFN concentration, within certain limits.

### 3.2. SPR Sensorgram of the Sample

**Figure 2.**
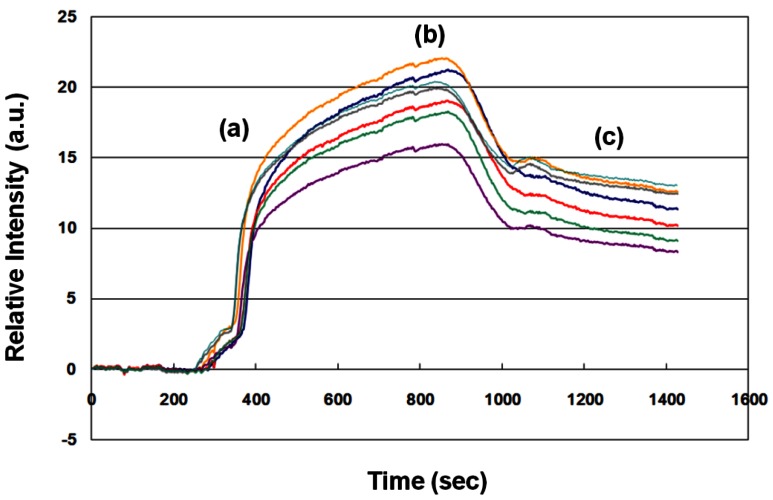
Sensorgram of a clinical sample from a pregnant woman at 33 gestational weeks. (**a**) Association, (**b**) Steady state, and (**c**) Dissociation of the antibody-antigen.

One example (no. 23 in Table 1) of an SPR sensorgram of a sample from a pregnant woman at 33 gestational weeks is shown in Figure 2. About 1,100 s elapsed from the beginning of antibody-antigen association to the end of antibody-antigen dissociation. Therefore, we evaluated the fFN concentration in clinical samples according to the average dynamic fitting curve. The average SPR relative intensity was 11.19 a.u. In this case, the uterine contraction duration/interval was 20 s/5 min, and the cervical os was not dilated by speculum examination. Hospitalization for tocolytic therapy was not suggested by the evaluation of obstetricians, and the woman did not deliver until 41 gestational weeks. The clinical result was compatible with the lower level of fFN measurement (<17 a.u.).

### 3.3. Analysis of Clinical Samples

Sixty-four samples were obtained. After excluding the cases of advanced cervical dilation (≥3 cm), rupture of amniotic membranes, significant vaginal bleeding, sexual intercourse within 24 h, multiple gestations, and prior tocolytic therapy in this pregnancy, 32 clinical samples were analyzed (Table 1). Preterm delivery usually ensues after advanced cervical dilation or rupture of amniotic membranes; fFN measurement is therefore not useful for these cases. Remarkable vaginal bleeding may be related to other obstetric problems such as placenta previa or abruptio placenta, which are the other important risk factors of preterm birth. In addition, testing a sample with amniotic fluid, blood, or semen may increase the possibility of false positive results.

The average detection weeks were 29.91 ± 4.08 (range 22 to 34 weeks), and delivery weeks were 35.56 ± 4.28 (range 22 to 41 weeks). No cervical dilation was found in 27 (84.38%) cases. Twenty-six (81.25%) of the pregnant women were admitted to the hospital for tocolytic therapy after the evaluation of obstetricians. Eleven (34.38%) of the 32 samples had an SPR intensity greater than or equal to 17 a.u., and 21 (65.62%) had an SPR intensity less than 17 a.u. No statistically significant differences were found between the two groups in detection weeks and cervical dilation. These results may have occurred because we excluded cases of advanced cervical dilatation from analysis. However, there were significant differences between the two groups in regular uterine contractions (duration ≥ 10 s and interval ≤ 3 min) (*p* = 0.040), hospitalization for tocolysis (*p* = 0.049), and delivery weeks (*p* = 0.043) (Table 2). These findings agree with previous studies performed using the fFN ELISA test [12,13,19–21]. In our study, regular uterine contractions were noted in six (54.55%) of the 11 pregnant women with SPR intensity greater than or equal to 17 a.u., but only in four (19.05%) of the 21 pregnant women with SPR intensity less than 17 a.u. (*p* = 0.040). Uterine contractions may cause placental shearing, which induces separation of the chorion layer from the decidual layer of the uterus, and then fFN is released into the cervix and vagina. Hospitalization for tocolysis occurred in all (100%) of the 11 pregnant women with higher SPR intensity, but only 15 (71.43%) of the 21 pregnant women with lower SPR intensity (*p* = 0.049) were hospitalized for tocolysis. Clinically, after excluding medical or surgical problems (such as acute gastroenteritis or appendicitis) and other potential obstetric causes of preterm labor (such as abruptio placenta), obstetricians evaluate the severity of uterine contractions and/or the progression of cervical dilation to decide the necessity of hospitalization for tocolysis. Finally, we found that delivery occurred nearly four weeks earlier in the group with higher SPR intensity (33.00 ± 5.39 *vs.* 36.90 ± 2.90, *p* = 0.043). These results show that preterm delivery prediction by fFN detection can be accomplished using our chip, and the SPR-based biosensor is another choice for fFN measurement.

### 3.4. ROC Curve of Higher SPR Intensity

**Figure 3.**
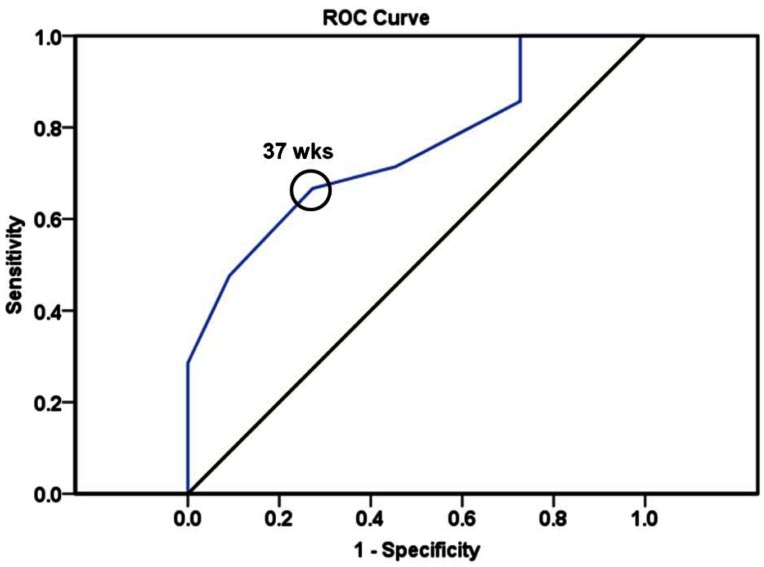
Receiver-operating characteristic (ROC) curve for predicting the delivery weeks for the group with higher SPR relative intensity. The optimum cut-off point (open circle) was defined as the closest point on the ROC curve to the point (*x, y*) = (0, 1), where *x* = 1 − specificity and *y* = sensitivity.

Figure 3 shows an ROC curve and the optimum cut-off point of predicting delivery weeks in the group with SPR relative intensity greater than or equal to 17 a.u. The AUC was 0.751 for delivery weeks (*p* = 0.021, 95% CI 0.582–0.920). On constructing the ROC curve, we revealed that a cut-off value of 37 gestational weeks in the group with higher SPR intensity provided the best sensitivity (66.7%) and specificity (72.7%) for predicting risk of preterm birth. These results agree with the definition of preterm birth as delivery at less than 37 gestational weeks [1].

### 3.5. SPR-Based Biosensors *versus* ELISA

Many methods have been used to detect the concentration of biomarkers. The most prevalent method for the quantitative detection of molecules in biological samples is ELISA. The principle of ELISA is based on two main procedures: (1) utilizing a primary antibody to bind the antigen of interest; (2) labeling a secondary enzyme-linked antibody to produce a quantitative alteration for measurement [27]. ELISA has many advantages, such as widely available facilities, reagents with long shelf lives, and a lack of radioactive dangers during labeling or waste disposal (compared with radioimmunoassay). However, ELISA also has some disadvantages, such as its time-consuming nature (more labeling and detection steps), its more expensive kits, the effects of biological matrices on enzyme activity, and the decay of color signal intensity. Further technologies have been developed to overcome these drawbacks; examples include mass spectrometry, flow cytometry, SPR, and recent immunomagnetic reduction [28].

In contrast to ELISA, the SPR-based biosensor is real-time, label-free, fast, and low-cost. It can quantitatively measure molecules of interest without the need for secondary antibodies to generate signals. Furthermore, in real-time analysis, SPR-based biosensors can illustrate association and dissociation phases and determine the affinity and kinetics of molecular interaction [29–31]. Many studies have also demonstrated that the SPR-based biosensor is more sensitive than ELISA and has a similar specificity [32–36]. In Taiwan, the commercial fFN ELISA kit is not widely used because of its higher cost. However, the AUC, sensitivity, and specificity of our data were similar to that of previous studies with ELISA [21,37]. In the cases that did not fall in the ROC curve, we additionally assessed the cervical length under ultrasound to predict the risk of preterm labor [38]. According to the instructions of the fFN ELISA kit, the test results are generally available within 24 to 48 h after sample collection. Our SPR-based biosensor allows fast measurement in about 2 h. This time advantage is crucial for making decisions in urgent situations. Furthermore, SPR-based biosensor platforms to perform simultaneous multi-target measurement have become available in recent years [39,40]. Thus it is possible to detect the above-mentioned biomarkers of preterm labor simultaneously, such as IL-6, MCP-1, phIGFBP-1, and MMP-8 [6–10], improving the accuracy of predicting the risk of preterm delivery. The SPR-based biosensor is a tool with great potential, and recent studies have focused on the development of high throughput and microinstrumentation of biosensors [41–44], further increasing its potential.

## 4. Conclusions

In this study, we constructed a fitting curve of standard fFN concentrations and compared standards with clinical samples. Our results show that SPR-based biosensors can quantitatively analyze fFN concentrations. The SPR-based biosensor is a promising method with quantitative detection, high sensitivity, high specificity, easy operation, and low cost. Our findings revealed the potential of the SPR-based biosensor in predicting the risk of preterm birth; the SPR-based biosensor may be a good alternative to conventional methods such as ELISA. Using SPR-based biosensors, we can diagnose preterm labor more accurately. A positive fFN test result can alert us to treat patients as soon as possible, such as by administering tocolytic therapy or steroids for fetal lung maturity and by referring patients to the tertiary medical center for better neonatal care, in order to decrease the incidence of perinatal morbidity and mortality. A negative fFN test result may reduce the over-diagnosis of preterm labor and avoid unnecessary medical intervention.

## Figures and Tables

**Table 1.** Patient characteristics.

**Patient No.**	**Detection weeks**	**SPR relative intensity (a.u.)**	**Cervical dilatation (cm)**	**Uterine contractions (duration/interval)**	**Hospitalization for tocolysis**	**Delivery weeks**
1	30	0.93	0	20″/3–5′	No	40
2	34	9.85	0	5″/2–3′	Yes	38
3	23	8.86	0	20″/5–6′	No	39
4	25	8.58	0	15″/4′	Yes	38
5	23	5.93	0	irregular	Yes	35
6	26	29.97	0	20–30″/2–3′	Yes	27
7	32	4.33	0	irregular	Yes	37
8	25	23.34	0	15″/5–8′	Yes	34
9	30	20.63	2	20″/3′	Yes	38
10	33	3.49	0	20″/5–6′	Yes	36
11	32	32.72	0	10″/2–3′	Yes	37
12	34	5.76	0	irregular	No	38
13	33	7.33	1	20″/3–6′	Yes	33
14	32	10.42	2	10″/2–3′	Yes	32
15	32	25.31	0	10″/6–7′	Yes	36
16	33	15.99	0	10″/2–3′	No	37
17	25	47.95	0	1′/6–7′	Yes	26
18	31	13.01	0	20″/8′	Yes	37
19	33	15.39	0	15″/3–6′	Yes	34
20	26	31.44	1	15″/5–8′	Yes	35
21	34	15	0	15′/3–6′	No	38
22	33	10.68	0	20″/2–3′	Yes	37
23	33	11.19	0	20″/5′	No	41
24	28	13.15	0	10″/6–7′	Yes	30
25	33	8.46	0	10″/2–5′	Yes	35
26	34	13.51	0	20″/5′	Yes	40
27	22	26.01	0	10″/3′	Yes	22
28	22	10.63	0	irregular	Yes	40
29	33	17.99	0	20″/5–6′	Yes	36
30	33	42.19	0	10″/3′	Yes	35
31	27	7.08	0	5–10″/2–3′	Yes	40
32	33	18.02	1	10–15″/2–3′	Yes	37

**Table 2.** Demographic data between SPR relative intensity ≥17 a.u. and <17 a.u.

**SPR relative intensity**	**≥17 a.u.**	**<17 a.u.**	***p* value**
No.	11 (34.38%)	21 (65.62%)	
Detection weeks	28.82 ± 4.07	30.48 ± 4.07	0.282
Cervical dilatation (cm)	0.36	0.14	0.290
Regular uterine contractions (≥10″/≤3′)	6 (54.55%)	4 (19.05%)	0.040
Hospitalization for tocolysis	11 (100%)	15 (71.43%)	0.049
Delivery weeks	33.00 ± 5.39	36.90 ± 2.90	0.043
